# Human-like Dexterous Grasping Through Reinforcement Learning and Multimodal Perception

**DOI:** 10.3390/biomimetics10030186

**Published:** 2025-03-18

**Authors:** Wen Qi, Haoyu Fan, Cankun Zheng, Hang Su, Samer Alfayad

**Affiliations:** 1 School of Future Technology, South China University of Technology, Guangzhou 511442, China; wenqi@scut.edu.cn (W.Q.);; 2 The IBISC Laboratory, UEVE, University of Paris-Saclay, 91000 Evry, France; samer.alfayad@univ-evry.fr

**Keywords:** human-robot interaction, reinforcement learning, tactile feedback, multimodal perception, hand gesture recognition

## Abstract

Dexterous robotic grasping with multifingered hands remains a critical challenge in non-visual environments, where diverse object geometries and material properties demand adaptive force modulation and tactile-aware manipulation. To address this, we propose the Reinforcement Learning-Based Multimodal Perception (RLMP) framework, which integrates human-like grasping intuition through operator-worn gloves with tactile-guided reinforcement learning. The framework’s key innovation lies in its Tactile-Driven DCNN architecture—a lightweight convolutional network achieving 98.5% object recognition accuracy using spatiotemporal pressure patterns—coupled with an RL policy refinement mechanism that dynamically correlates finger kinematics with real-time tactile feedback. Experimental results demonstrate reliable grasping performance across deformable and rigid objects while maintaining force precision critical for fragile targets. By bridging human teleoperation with autonomous tactile adaptation, RLMP eliminates dependency on visual input and predefined object models, establishing a new paradigm for robotic dexterity in occlusion-rich scenarios.

## 1. Introduction

The replication of human dexterity in robotic manipulation remains an open challenge, particularly in non-visual environments where objects with diverse geometries and material properties demand adaptive grasping strategies. While recent advances in robotic hand design have achieved remarkable morphological similarity to human hands [1,2], existing control paradigms struggle to handle the infinite physical permutations encountered in real-world manipulation tasks. Traditional model-based approaches [3,4] rely on precise mathematical formulations of hand-object interactions, yet their effectiveness diminishes when confronted with unstructured scenarios requiring real-time tactile adaptation. This fundamental limitation becomes particularly acute when manipulating objects that demand continuous force modulation without visual feedback—a capability intrinsic to human manipulation but elusive in robotic systems. Emerging data-driven solutions [5,6,7] show promise in bridging this gap, yet often neglect the biological intelligence encoded in human sensorimotor coordination.

Recent advances in reinforcement learning (RL) have propelled human-robot interaction (HRI) toward resolving two interdependent challenges: dynamic adaptation to physiological variability and safety-guaranteed coordination in unstructured environments [8]. Central to this progress are human-in-the-loop RL frameworks that empirically optimize collaborative parameters through iterative physical interactions [9], effectively bridging deterministic control policies with stochastic human behaviors. Building on these foundations, hierarchical multi-agent architectures now integrate adversarial training and causal reasoning to disentangle intent ambiguities during complex manipulation tasks [10], while inverse RL methodologies enable robots to infer latent reward functions from expert demonstrations, replicating human-like force modulation without tactile feedback [11]. In safety-critical domains, hybrid paradigms combining augmented reality with deep RL achieve real-time collision prediction through multimodal sensor fusion [12], complemented by intrinsic reward mechanisms that autonomously balance task efficiency and risk mitigation [13]. Despite these innovations, current RL-driven HRI systems predominantly prioritize task-level metrics over the embodied cognitive principles governing human dexterity [14], limiting their ability to generalize across non-visual manipulation scenarios requiring symbiotic sensorimotor intelligence.

With increasing task complexity, the integration of multifinger dexterous robotic hands for grasping various objects has driven advances in data set availability and algorithmic sophistication, with recent studies showcasing the efficacy of large-scale reinforcement learning (RL) in addressing these challenges [15,16,17]. Unlike traditional deep learning methods constrained by task-specific limitations, RL thrives in dynamic, unstructured environments, providing remarkable adaptability and performance, especially in navigating the complexities of volatile grasping scenarios, where unsupervised strategies like RL offer significant innovative potential [18]. Model-free RL approaches, though promising, involve challenges such as precise reward configuration, prolonged iterative training, and substantial computational resources, often necessitating industrial-scale systems, thus complicating the application of dexterous manipulation to real-world problems [4]. These constraints raise a pivotal question: can dexterous behaviors be learned with greater sample efficiency, reducing computational and time demands while preserving or enhancing performance, thus unlocking new avenues for efficient and versatile solutions in complex robotic manipulation tasks?

Previous research has explored a wide range of gesture recognition models to facilitate human-robot teleoperation, utilizing various data sources such as skeletal motion, EMG, and joint angle information [19]. A notable approach integrated sensor fusion of hand kinematics for efficient control of underactuated bionic hands with minimal input data, while another innovative strategy employed a multilayer LSTM-RNN to classify hand gestures from depth vision data, highlighting the potential for advanced gesture recognition [20]. Although these models are effective, they typically rely on extensive data collection and preprocessing, without being tested in conjunction with real robotic systems to address potential issues that may arise in practical implementation. Reinforcement learning-based approaches offer a compelling alternative, enabling the system to learn from predefined rules, reducing dependence on large datasets, and providing a flexible framework for adapting to complex teleoperation tasks.

**Figure 1:**
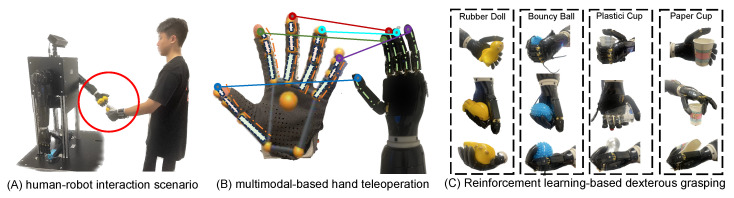
The presented graph illustrates the three-layered framework of our anthropomorphic dexterous grasping system.

While recent advances in robotic manipulation have improved grasping reliability, existing systems still struggle with adaptive force control when handling unknown objects in unstructured environments—particularly in integrating human demonstration with autonomous tactile adaptation. This paper introduces the Reinforcement Learning-Based Multimodal Perception (RLMP) framework, illustrated in Figure 1, which aims to enhance the effectiveness of dexterous grasping through multimodal hand teleoperation and reinforcement learning-based modeling. (a) Human-Robot Interaction: The robotic hand’s functionality is evaluated in scenarios such as stabilizing a paper cup or performing clinking gestures, showcasing its interactive dexterity. (b) Teleoperation through Multimodal Inputs: A custom glove transmits precise data by modeling and processing the Euler angles of finger joints, enabling accurate guidance of the robotic hand’s movements. (c) Reinforcement Learning for Grasp Refinement: Adaptive reinforcement learning techniques are employed, leveraging diverse objects to iteratively enhance grasping efficiency and versatility. By integrating multiple sensory modalities, the RLMP framework facilitates more precise and adaptive grasping in complex and dynamic environments. The key contributions of this work include:

- A human-robot co-learning framework (RLMP) that synergizes novice-friendly teleoperation with reinforcement learning, enabling robots to acquire adaptive grasping strategies for objects with diverse geometries and material properties, without requiring pre-defined object models or visual feedback.
- A five fingers tactile-centric RL architecture that seamlessly correlates finger kinematics with tactile sensory input, eliminating the necessity for prior domain knowledge.
- An innovative tactile recognition method that utilizes deep convolutional networks to extract material specific features from high-dimensional pressure matrices, achieving visual object recognition without supervision or manual labeling, which is crucial for adaptive grasping in visually blurry environments.

This study introduces the RLMP framework to address challenges in dexterous robotic grasping. Section 2 review previous advancements in robotic hand grasping, emphasizing model-based and RL approaches, highlight the integration of multimodal perception in teleoperation systems, and examine RL applications in human-robot interaction (HRI). Section 3 details the framework, including mapping glove movements to robotic hand actions for intuitive teleoperation, RL-based modeling to associate finger kinematics with tactile feedback, and the deployment of a DCNN for robust object recognition. In Section 4, comprehensive real-world tests demonstrate the effectiveness of the RLMP framework in achieving precise, contact-intensive grasps and accurate object identification in diverse scenarios. Finally, Section 5 highlights the significant contributions of the framework to improving dexterous robotic manipulation through multimodal perception and RL, offering a promising foundation to advance HRI applications in complex environments.

## 2. Related Works

The functional domains of robotic hands have been extensively explored in contemporary research, with a primary focus on the execution of intricate movements and tasks, as well as the precise mechanisms underlying object grasping and manipulation. The foundation of the proposed system draws on pivotal advances in the fields of dexterous grasping, teleoperation, and reinforcement learning. This section provides a concise synthesis of prior studies that form the contextual basis for the present investigation.

### 2.1. Dexterous Grasping via Robotic Hand

The pursuit of anthropomorphic dexterity in multi-fingered robotic grasping has driven innovations across domains ranging from precision manufacturing to teleoperated surgical systems. Recent advances in semantic-aware grasp synthesis leverage point cloud representations to encode object geometry and material properties, enabling robots to approximate human-like grasping adaptability under partial visual observations [21,22]. Particularly noteworthy are frameworks such as pregrasp-informed manipulation (PGDM), which circumvent the need for task-specific configurations by dynamically optimizing contact points through self-supervised learning, thereby enhancing generalization across unseen object categories [23]. Despite these methodological advancements, fundamental limitations persist across three critical fronts: the curse of dimensionality in reinforcement learning policy optimization, mechanical compromises between actuation speed and finger flexibility, and perceptual fragility during multi-directional target engagement. For instance, while generalized modeling approaches have improved rotational motion precision through kinematic constraints [4], they struggle to synchronize force modulation with trajectory planning in unstructured environments [24]. These unresolved challenges highlight the pressing need for control paradigms that holistically integrate biological principles with data-driven adaptability.

### 2.2. Multimodal Perception-Driven Teleoperation

The evolution of dexterous teleoperation systems increasingly relies on synergistic integration of heterogeneous sensor modalities to decode human manipulation intent [25]. Central to these efforts is the dual sensing paradigm: intrinsic measurements through position and bend sensors capture kinematic hand configurations, while extrinsic feedback from joint torque and tension sensors quantifies interaction dynamics, enabling comprehensive reconstruction of both positional and force-related manipulation states [26]. Complementing these approaches, haptic interfaces combined with electromyography (EMG) sensors establish bidirectional human-robot communication channels—translating muscle activation patterns into robotic force modulation while providing tactile feedback to operators [27]. However, prevailing methodologies exhibit two systemic limitations: (1) the persistent segregation of unimodal data streams (e.g., visual vs. proprioceptive) during perception fusion [28], and (2) the architectural decoupling between gripper hardware design and adaptive learning algorithms. This fragmentation fundamentally constrains the development of truly embodied teleoperation systems capable of simultaneous multimodality perception and dynamic tool-environment adaptation.

### 2.3. RL-Based HRI

The ability of a robotic system to securely grasp objects is vital for the successful execution of complex manipulation tasks. Over the years, a multitude of approaches grounded in deep learning have been proposed to address this challenge [29]. For instance, it framed grasping tasks as regression problems and leveraged the capabilities of Convolutional Neural Networks (CNNs) to tackle them. Although these vision-based models exhibit robust generalization in various grasping scenarios, their susceptibility to collisions often hampers their efficacy [30]. This limitation becomes particularly evident in tasks that require the manipulation of objects in close proximity, where the likelihood of collisions increases significantly [31].

However, RL aims to derive optimal policies that maximize the cumulative expected rewards. Recent advances in this domain have introduced RL-based frameworks specifically designed for dexterous grasping. These frameworks utilize multimodal grippers to streamline actions, enhancing efficiency in handling intricate manipulation tasks [28]. By integrating visual and tactile feedback, RL-based systems enable agents to continually gather new sensory data, facilitating dynamic refinement of their operational strategies in real time.

Unlike conventional deep learning approaches, which often depend on task-specific constraints and predefined conditions, RL methods exhibit significant potential for achieving superior performance in dexterous grasping. This advantage is particularly evident in dynamic and unstructured environments, where adaptability is crucial. The following sections of this paper provide an in-depth exploration of RL frameworks, highlighting their applicability and advantages in the context of advanced grasping tasks.

## 3. Method of RL-Based Multimodal Perception

**Figure 2:**
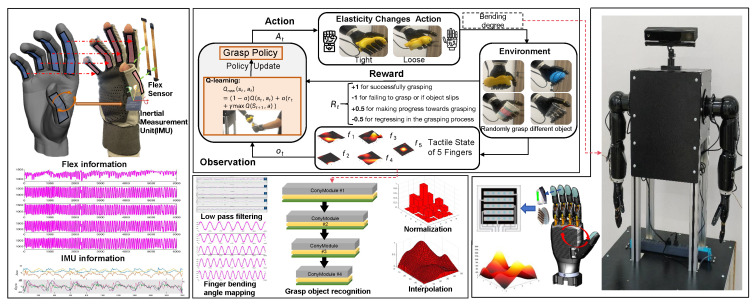
RLMP framework overall structure diagram, human-like dexterous grasping is realized through a series of interconnected processes.

The RLMP framework encapsulates a human-robot interaction process structured into three pivotal phases (see Figure 2): Initially, multimodal teleoperation is achieved through a sensorized glove that captures human hand kinematics via inertial measurement units and flexion sensors, with the acquired data being processed by a pose detection algorithm to generate robotic control signals through kinematic retargeting. Subsequently, reinforcement learning optimizes dexterous grasping by integrating tactile feedback matrices from 25-element pressure sensors with finger joint proprioception, where a Q-learning policy iteratively refines grasp stability through reward maximization based on object displacement minimization and contact force regulation. Finally, tactile patterns from historical grasp trials train a deep convolutional neural network classifier that enables real time object recognition by identifying material-specific pressure signatures, thereby closing the perception-action loop. This tripartite architecture establishes a bidirectional mapping between human demonstration and robotic execution while enabling adaptive grasping through continuous tactile learning.

### 3.1. Robotic Hand Mapping

**Figure 3:**
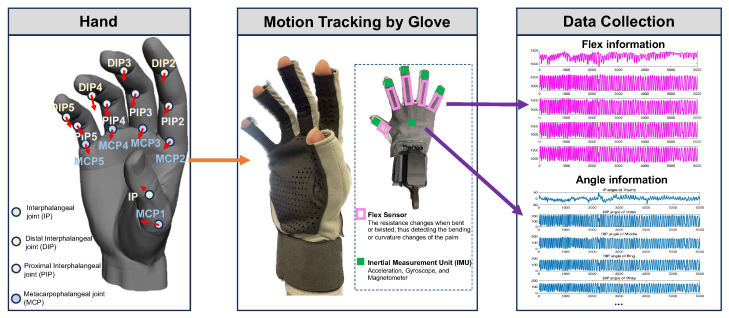
Introduction of palm structure and data glove perception information.

For the robotic transposition of human hand movements, we utilize a glove sensor system that captures both curvature and Euler angles. As illustrated in Figure 3, the glove is equipped with six inertial measurement units (IMUs) and five flex sensors, which collectively record acceleration data $A_{i}=\left[a_{xi},a_{yi},a_{zi}\right]$, gyroscopic measurements $G_{i}=\left[\omega_{xi},\omega_{yi},\omega_{zi}\right]$, and flex values $F_{i}$ for i∈{1,⋯,6}, corresponding to the back of the palm and each of the fingers. To accommodate the inherent variability in human joint postures, it is crucial to perform a localized mapping of the IMU data for each distal interphalangeal joint (DIP). This process involves transforming the data from a global coordinate system to a palm-centered reference frame, ensuring a more accurate representation of the movements of the hands [32,33].

The Kalman filter operates directly on raw gyroscopic ($A_{i}$) and accelerometric ($G_{i}$) measurements, producing denoised $\hat{A_{i}}$ and $\hat{G_{i}}$ sequences [34].

(1) $$\begin{array}{cc} \mathrm{State}\;\mathrm{vector}:\; & X_{k}={\left[\begin{array}{c} \omega_{xi},\omega_{yi},\omega_{zi},a_{xi},a_{yi},a_{zi} \end{array}\right]}_{k|k-1}^{⊤}\\ \mathrm{State}\;\mathrm{transition}:\; & X_{k}=FX_{k-1}+w_{k},\;F=\left[\begin{array}{cc} I_{3} & \Delta t\cdot I_{3}\\ 0_{3} & I_{3} \end{array}\right]\\ \mathrm{Measurement}:\; & Z_{k}=HX_{k}+v_{k},\;H=I_{6} \end{array}$$

where Δt denotes the sampling interval, with $w_{k}∼N\left(0,Q\right)$ and $v_{k}∼N\left(0,R\right)$ representing process and measurement noise respectively. The filter recursively computes:

(2) $${\hat{X}}_{k}={\hat{X}}_{k|k-1}+K_{k}\left(Z_{k}-H{\hat{X}}_{k|k-1}\right)$$

where the Kalman gain $K_{k}$ minimizes the estimation error covariance.

To mitigate orientation-induced signal artifacts caused by arbitrary hand positions during operation, we first establish a unified coordinate framework through sensor alignment. This critical preprocessing step eliminates waveform distortions and signal level discrepancies that would otherwise propagate through subsequent calculations when the hand assumes non-canonical orientations. Initially, the orientation of the IMU in the dorsal hand is consistent with the global system, implying that the rotation matrix *R* defaults to the identity matrix *I*.

(3) $$R=\left[\begin{array}{ccc} 1 & 0 & 0\\ 0 & 1 & 0\\ 0 & 0 & 1 \end{array}\right]$$

For each sampling time step Δt, rotation matrix *R* with:

(4) $$\begin{array}{cc} R_{i}^{*} & =R_{i}\cdot \exp\left(G_{i}\cdot \Delta t\right)\\ \; & =R_{i}\cdot \left[e^{\omega_{x}i\Delta t},e^{\omega_{y}i\Delta t},e^{\omega_{z}i\Delta t}\right] \end{array}$$

Subsequently, determine the relative rotation matrix $R_{ct_{i}}$ from the center of the palm to the five fingers. Transform the acceleration, angular velocity, and gravitational acceleration *g* from the global to the local coordinate system.

(5) $$\left\{\begin{array}{c} R_{c_{i}}=R_{0}^{T}\cdot R_{i}\\ A_{c_{i}}=R_{0}^{T}\cdot A_{i}\\ G_{c_{i}}=R_{0}^{T}\cdot G_{i}\\ g^{\prime }=R_{0}^{T}\cdot g \end{array}\right.$$

Therefore, the DIP angles could be calculated as:

(6) $$\begin{array}{cc} \; & a_{linear_{i}}=\sqrt{a_{xi}^{2}+a_{yi}^{2}+a_{zi}^{2}-{g^{\prime }}^{2}}\\ \; & \theta_{i}=arctan\left(\frac{a_{linear_{i}}}{r}\right) \end{array}$$

where *r* is the radius of the glove’s bending axis.

To address the limitations of single-sensor mapping, we consider a Multimodal mapping strategy to processes IMU orientations $\theta_{i}$ and flex values $F_{i}$ through coordinate transformation and confidence-aware fusion [20,35]:

(7) $$\theta_{\mathrm{fused}}=w_{\mathrm{imu}}\cdot \theta_{\mathrm{imu}}+w_{\mathrm{flex}}\cdot F_{i}$$

where the adaptive weights are computed from sensor variances:

(8) $$w_{\mathrm{imu}}=\frac{\sigma_{\mathrm{flex}}^{2}}{\sigma_{\mathrm{imu}}^{2}+\sigma_{\mathrm{flex}}^{2}},\;w_{\mathrm{flex}}=1-w_{\mathrm{imu}}$$

with $\sigma_{\mathrm{imu}}^{2}=0.8$ and $\sigma_{\mathrm{flex}}^{2}=0.3$ obtained through 200 static calibration trials.

Kinematic Mapping:

(9) $$q_{\mathrm{robot}}=K\cdot \theta_{\mathrm{fused}}+b$$

where the scaling matrix K=diag([1.05,0.97,1.02,1.1,0.95]) and offset b are derived through least-squares fitting on calibration trajectories.

### 3.2. Reinforcement Learning Framework

Our reinforcement learning framework employs a multimodal state representation that synergistically integrates proprioceptive, tactile, and object-aware information. As depicted in Figure 2, the learning process operates through continuous interaction with the environment, where the agent’s policy π:S→A maps observed states to joint torque adjustments. Departing from conventional single-modality approaches, we formulate the Markov Decision Process (MDP) with three complementary state components and a constrained action space, specifically designed for dexterous grasping scenarios. The key innovations include: (1) hierarchical abstraction of high-dimensional tactile data, (2) safety-aware action constraints to prevent mechanical overload, and (3) an adaptive reward mechanism balancing grasp stability and force minimization.

**Figure 4:**
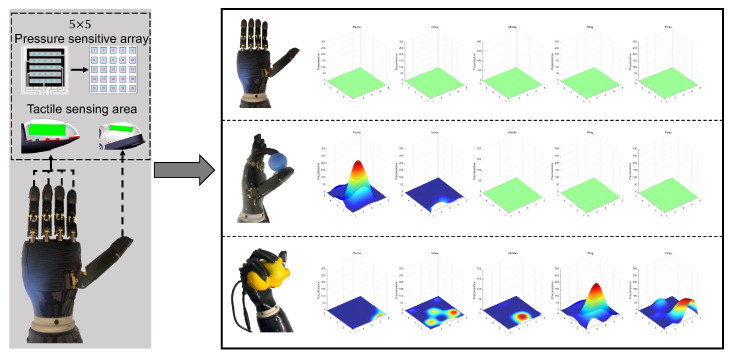
Introduction of tactile perception of dexterous hands and data states under different grasping states.

The Markov Decision Process (MDP) is formally defined as follows: State Space S:

- Joint state:
  (10) $$s_{j}=\left[\theta_{1},\omega_{1},\ldots ,\theta_{5},\omega_{5}\right]\in R^{10}$$
  where $\theta_{i}$ and $\omega_{i}$ represent the angular position (rad) and velocity (rad/s) of the *i*-th finger joint.
- Tactile state:
  (11) $$s_{t}=\mathrm{flatten}\left(M_{5\times 5}\right)\in R^{25}$$
  converting the pressure matrix *M* into a vector through row-wise flattening. Tactile perception of dexterous hands and data states under different grazing states as shown in Figure 4).
- Object properties:
  (12) $$s_{o}=\left[m,k\right]\in R^{2}$$
  where *m* denotes mass (kg) and *k* represents stiffness coefficient (N/m).

Action Space A:

(13) $$a=\left[\tau_{1},\ldots ,\tau_{5}\right]\in R^{5},\;\Delta \tau_{i}\in \left[-0.2,0.2\right]\;N\cdot m$$

with the safety constraint:

(14) $$\sum_{i=1}^{5}\left|\tau_{i}\right|\leq 1.5\;N\cdot m$$

The composite reward function combines three essential objectives:

(15) $$r\left(s,a\right)=w_{1}\cdot e^{-\beta |\Delta p|}-w_{2}\cdot \sum \left|f_{i}\right|+w_{3}\cdot I_{\mathrm{stable}}$$

- Δp: Position displacement error (mm) between current and target grasp
- $f_{i}$: Pressure deviation at *i*-th tactile element from desired range
- $I_{\mathrm{stable}}$: Time-dependent stability bonus (0.1 per second)

The weighting coefficients are empirically set as:

(16) $$w_{1}=2.0,\;w_{2}=0.5,\;w_{3}=0.3,\;\beta =0.1$$

Q-learning operates as an offline policy optimization method, whereas SARSA serves as an online policy optimization technique, inherently managing the trade-off between exploration and exploitation throughout the learning phase and adhering to the prevailing policy. Within the system, three distinct grasping states are delineated: standard grasp, overly loose grasp, and overly tight grasp. During teleoperation and object perception, the tactile feedback thresholds are fine-tuned to achieve standard grasping for various objects [36].

For Q-Learning, the update procedure is given as:

(17) $$\begin{array}{cc} Q_{new}\left(s_{t},a_{t}\right)= & \left(1-\alpha \right)Q\left(s_{t},a_{t}\right)\\ \; & +\alpha (r_{t}+\gamma max_{a}Q\left(s_{t+1},a\right)) \end{array}$$

For SARSA:

(18) $$\begin{array}{cc} Q_{new}\left(s_{t},a_{t}\right)= & \left(1-\alpha \right)Q\left(s_{t},a_{t}\right)\\ \; & +\alpha (r_{t}+\gamma Q\left(s_{t+1},a_{t+1}\right)) \end{array}$$

The subsequent state and action, $s_{t+1}$ and $a_{t+1}$, are derived via the ϵ-greedy method. Before running the update, it is essential to define the optimal hyperparameters: learning rate α, ϵ in ϵ-greedy strategy and reward matrix *R*. This initialization promotes the integration of recent data with obsolete insights, improving dynamic learning. Hyperparameter selection plays a pivotal role in balancing immediate versus future returns in the reinforcement learning paradigm. In this context [37,38], decisions are temporally based on the agent’s discrete choices of states *s* and actions *a* at specific moments, namely *t* and t+1.

### 3.3. Object Recognition

**Figure 5:**
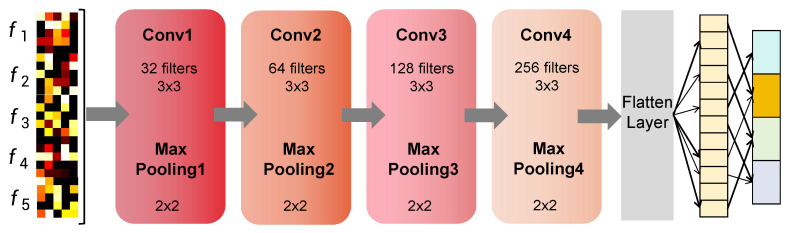
Structure diagram of classifier based on tactile data.

To address the challenge of tactile feedback-driven object recognition in dexterous robotic manipulation, we propose a Tactile-Driven DCNN architecture for processing pressure matrices (as shown in Figure 5). The network’s input is a 25×5 tactile pressure matrix, representing the concatenated result of the tactile pressure measurement matrices from five fingers.

The architecture begins with a feature extraction module comprising four sequential convolutional layers. Each layer employs 3×3 convolutional kernels with ReLU activation and batch normalization, progressively increasing the number of filters (32,64,128,256) to hierarchically encode multi-scale spatial patterns. A 2×2 max-pooling operation follows every convolutional layer to reduce feature map dimensions while preserving dominant activation patterns. To ensure robust generalization, batch normalization is applied after each convolution to stabilize training dynamics, and a dropout layer (rate 0.5) is introduced after the final pooling stage to mitigate overfitting.

The classification module consists of a fully connected layer with 512 ReLU-activated units, which processes the flattened output from the convolutional base, followed by an output layer with softmax activation for multi-class probability estimation [39]. The model is trained end-to-end using the Adam optimizer with an initial learning rate of $1\times 10^{-2}$, mini-batches of 50 samples, and a dropout rate of 0.001 applied during optimization. Training spans 300 epochs, with the mean squared error (MSE) serving as the loss function to minimize divergence between predicted and ground-truth class vectors in the hierarchical feature space. This architecture balances spatial feature extraction and regularization strategies to ensure adaptability in dynamic tactile perception tasks.

## 4. Experimental Evaluation and Discussion

### 4.1. HRI Platform

**Figure 6:**
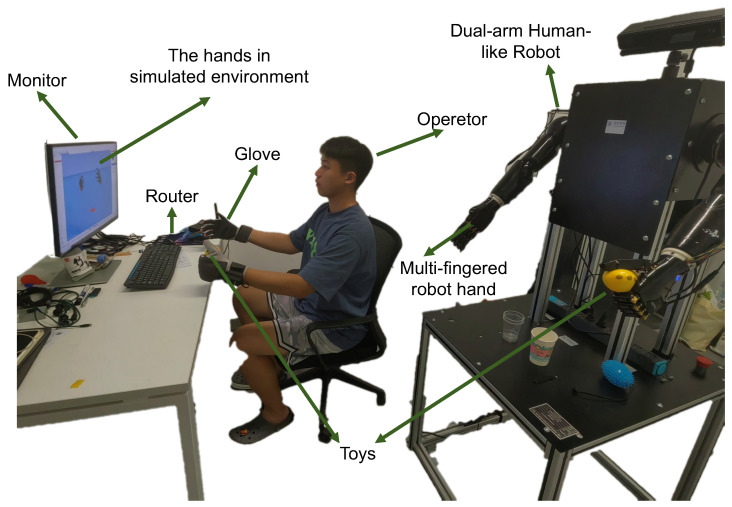
Overview of the designed Human Robot Teleoperation system.

The experimental platform, as depicted in Figure 6, represents a comprehensive humanoid arm robot system, which is composed of three key components:

- Humanoid Dual-arm Robot: The core of the experimental setup is a dual-arm humanoid robot, consisting of a fixed base, anthropomorphic mechanical arms, a tactile five-finger dexterous hand, a depth vision unit, and a main control unit. The anthropomorphic mechanical arms feature shoulder joints with three rotational degrees of freedom (DoFs), enabling forward-backward swinging, inward-outward expansion, and rotational movements; the elbow has one pitch DoF for flexion-extension control, while the wrist integrates three rotational DoFs. The tactile five-finger dexterous hand comprises six DoFs in total, with each finger equipped with a single-axis linkage bending joint for grasping actions, and the thumb additionally provided with a flexion-extension DoF to enable bidirectional motion capability. The fingertip area integrates a high-density 5×5 tactile sensor array, capable of detecting force interactions within a range of 0–5 N. Additionally, the depth vision module, located at the top of the robot, can acquire RGBD view data within its range, while the main control unit board, positioned at the center of the robot’s body, handles signal transmission and reception, performs related edge computing, and is equipped with connectivity options, including a standard serial port and a modern Wi-Fi interface.
- Custom Data Glove:Complementing the robotic system is a custom-designed data glove, aimed at providing real-time kinesthetic feedback of human hand movements. The glove embeds five inertial measurement units (IMUs) at the fingertips and one IMU at the back of the palm to track motion information, while five flexible bending sensors are installed in the finger sleeve areas to capture the bending state of each finger during movement. The glove is equipped with a state-of-the-art transceiver system, utilizing a router to synchronously and robustly receive signals from both gloves, enabling synchronized data collection at multiple frequencies (25 Hz, 50 Hz, and 100 Hz).
- Computational Apparatus: The system’s data analysis, real-time processing capabilities, and algorithmic workload are powered by a high-performance experimental PC equipped with an Intel i7 processor clocked at 2.80 GHz, complemented by 16 GB of RAM, enabling efficient multitasking and high data throughput. For GPU-intensive tasks and advanced simulations, the PC is also integrated with a state-of-the-art NVIDIA RTX 3070 Ti graphics processing unit.

### 4.2. Multimodal Hand-Robot Mapping Estimation

**Figure 7:**
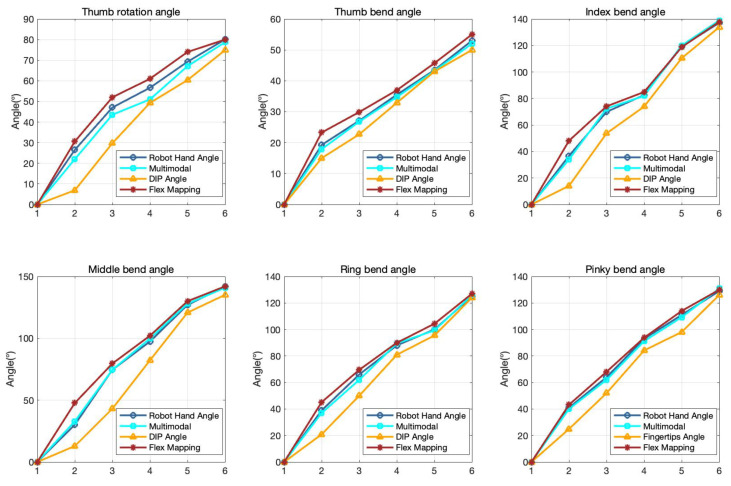
Comparison of angles in different ways of mapping from hand to manipulator joint angles.

To enable teleoperation of the robotic hand that closely emulates human gestures, the system integrates a sophisticated multisensor data fusion methodology. Central to this approach is a comparative study that critically evaluates the performance of the fusion technique against two alternative approaches: one based on controlling the DIP joint angles and the other focused on the flexural bending of the joints. The experimental results, visually presented in Figure 7, reveal an important observation: reliance on the angle control of the DIP joint alone leads to significant mapping errors, particularly when there are small variations in angle. This issue can be attributed to the fact that natural hand movements require the simultaneous adjustment of multiple joints, and subtle variations in DIP angles are often compounded by changes in adjacent joints, which are not considered in this approach. In contrast, the flexural bending method introduces challenges of its own, primarily due to the complex interdependencies among the various degrees of freedom in the robotic hand. The motion of a single joint can affect adjacent joints, creating difficulties in achieving effective alignment.

Table 1 provides a numerical analysis of the results. Compared to directly using DIP mapping or Flex sensor mapping, the multimodal fusion method significantly outperforms other techniques. Except for the more complex variations in the Thumb Rotation angle, the multimodal mapping achieves the highest accuracy in the bending error comparison across the five fingers. This approach mitigates mapping errors caused by sensor inaccuracies or mechanical interference, which are common in alternative methods. Moreover, it demonstrates exceptional accuracy in teleoperation of the dexterous hand, enabling precise replication of intricate manipulation tasks and complex gestures. By integrating multiple sensors, the system effectively transmits the operator’s intent to the robotic hand, thereby minimizing operational deviations and errors. These findings strongly support the effectiveness of the multimodal fusion method, highlighting its immense potential in advancing humanoid robotics and enhancing their practical applications [40,41].

### 4.3. RL Performance

Our system integrates an RL-driven modeling strategy to facilitate the autonomous acquisition of object-specific grasping techniques by the dexterous robotic hand. In this system, the mechanical arms are only controlled through a fixed program to assist the interaction between the five finger tactile dexterous hand and humans. In this setup, human operators remotely control the hand to interact with four distinct objects: the rubber doll, the bounce ball, the plastic cup, and the paper cup. The selection of these four objects is grounded in their inherent characteristics, which lead to distinct variations in the tactile pressure matrices observed during grasping. Both the doll and the ball are elastic objects that deform when subjected to pressure, but the irregular shape of the doll results in a markedly different deformation pattern and pressure distribution compared to the spherical ball. In contrast, plastic and paper cups are rigid objects that are not expected to undergo deformation under normal conditions, with any deformation signaling potential damage. These two materials, differing in composition, exhibit distinct hardness levels and surface smoothness, factors that directly influence grasping performance. In sum, these four objects, chosen for their diversity in material properties and shapes, serve as a robust test bed to evaluate the grasping capabilities of the robotic hand in various scenarios. By analyzing tactile pressure matrices during interactions with these objects, the system continuously refines its grasping methodologies, adapting to the distinct curvature characteristics of each object.

The experimental procedure follows a three-stage architecture of “demonstration imitation-online learning-tactile optimization” (under the framework of Figure 2), implemented as follows: First, eight volunteer operators wearing data gloves randomly performed multiple grasping demonstrations (over 10 trials) for four types of objects: rubber doll, elastic ball, plastic cup, and corrugated paper cup. During grasping, the multimodal mapping results from glove data were fed back to drive the five-fingered dexterous hand in synchronous motion to replicate identical postures, while sensing the 5×5 pressure distribution matrix from fingertip tactile sensors. The reinforcement learning module constructs an initial Q-table by integrating demonstration data through an ϵ-greedy strategy (exploration rate ϵ = 0.4). Thereafter, the online learning phase commences: employing a dynamic learning rate α (baseline value 0.2, adaptively adjusted with tactile pressure gradients) and multiple discount factors γ = 0.1, 0.3, and 0.5 to update Q-values based on real-time tactile feedback.

**Figure 8:**
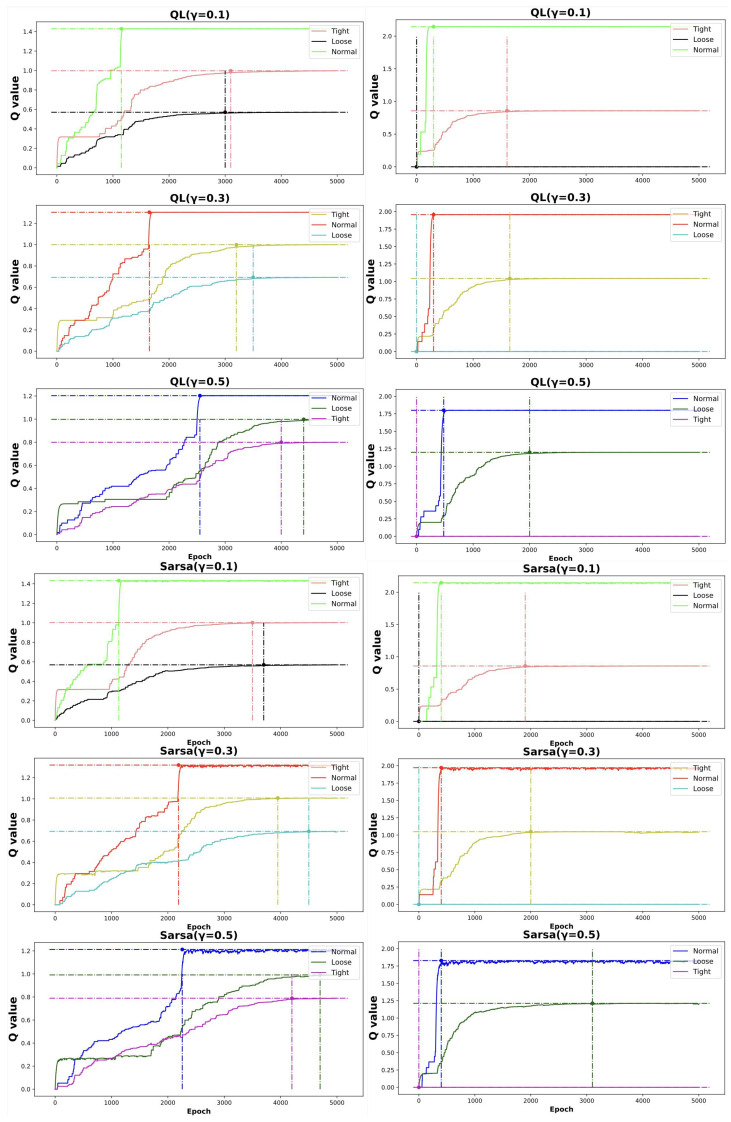
The quantitative analysis of changing Q values for Q-Learning (Left: Q = Q1, Right: Q = Q2) and SARSA (Left: Q = Q1, Right: Q = Q2) when γ=0.1,0.3,0.5. Epoch refers to the total number of complete traversal attempts.

The empirical results depicted in Figure 8 provide a comparative analysis of Q-learning and SARSA under varying discount factors (γ=0.1, 0.3, 0.5). The Q matrices are designed to encode distinct tactile-driven grasping states derived from the 5×5 pressure distribution matrices, where states such as ‘loose grasp’ and ‘stabilized grasp’ are mapped to reward structures. In particular, the matrices

$$Q_{1}=\left(\begin{array}{ccc} 2 & 1 & 3\\ 3 & 2 & 1\\ 1 & 3 & 2 \end{array}\right)$$

and

$$Q_{2}=\left(\begin{array}{ccc} 1 & -1 & 3\\ 3 & 1 & -1\\ -1 & 3 & 1 \end{array}\right)$$

capture distinct exploration-exploitation tradeoffs tied to γ values. The entries of the $Q_{1}$ and $Q_{2}$ were determined through a task-driven reward design combined with empirical calibration. Specifically, the values encode utility assessments for distinct tactile grasping states: high entries prioritize stable grasps, while penalties deter unsafe states. For γ=0.1, $Q_{1}$ prioritizes immediate rewards through rapid state transitions, aligning with the short-term focus required for initial contact phases. In contrast, $Q_{2}$’s penalty terms better support long-term stability goals when γ=0.5, albeit with delayed convergence. The interplay between γ and tactile-driven learning rates α reveals critical dynamics: Lower γ values complement adaptive α scaling (via pressure gradients), enabling fast adaptation to abrupt force changes (elastic objects). Higher γ (0.5) enhances coordination of multi-step force modulation (contact→stabilize→hold), as evidenced by reduced object slip events in post-optimization trials. SARSA’s inherent conservatism provides more stable policies for rigid objects, while Q-learning’s optimistic updates excel in exploratory grasping of deformable targets. These findings validate the framework’s capability to balance real-time responsiveness and strategic force planning through tactile-aware RL design.

### 4.4. Tactile-Driven Object Identification

**Figure 9:**
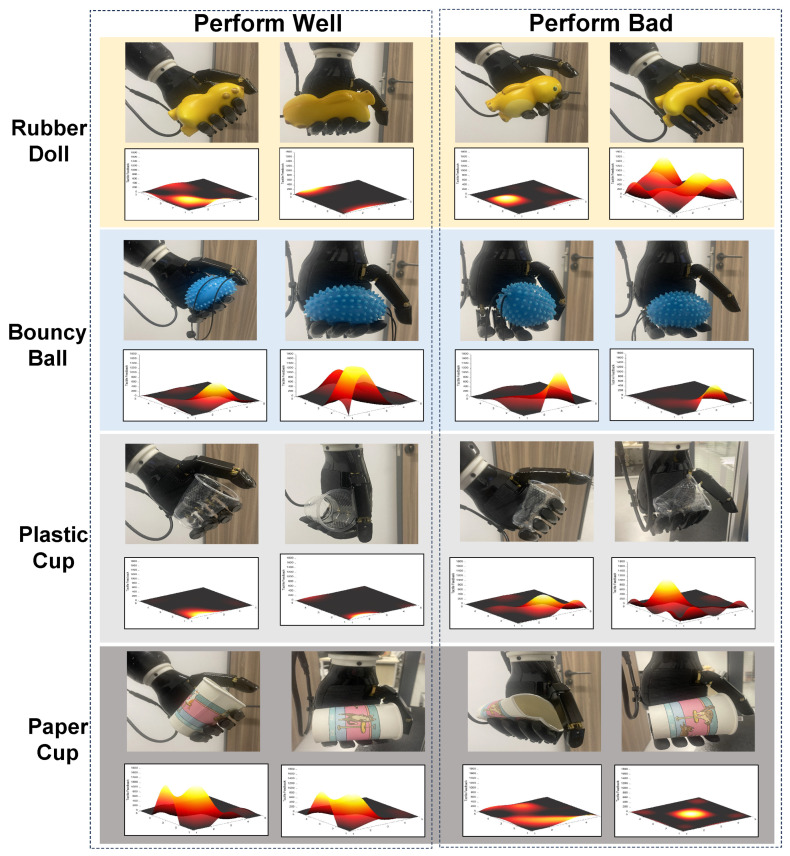
The efficacy of robotic hands in grasping diverse objects.

Figure 9 provides a meticulous evaluation of the robotic hand’s performance in a diverse set of object grasping scenarios through visual imagery and tactile matrices, while distributed matrices with moderate intensity typically signify successful grasping. Conversely, non-uniform distribution with pronounced concentration often suggests either overly firm or insufficiently secure grasps. The tactile sensors embedded in the hand play a pivotal role, serving as the essential instruments to capture critical metrics that are fundamental to assessing the effectiveness of the grasping process. Through a careful analysis of the tactile data, particularly examining the subtle variations in sensor responses that correspond to the distinct material properties of the objects, our study uncovers valuable insights into the various grasping strategies. This chapter underscores the significant role of grasping techniques in object recognition, with particular attention to the variance in grasp quality. Grasps that exhibit excessive force often lead to object deformation, which not only harms the object, but also hinders the ability of the robotic hand to maintain stable hold. In contrast, grasps that lack sufficient force risk instability, which can cause the object to slip from the grasp of the hand. In contrast, the ideal grasp demonstrates a harmonious balance, where force is applied with precision, ensuring both stability and effective manipulation without any detrimental effects on the object.

**Figure 10:**
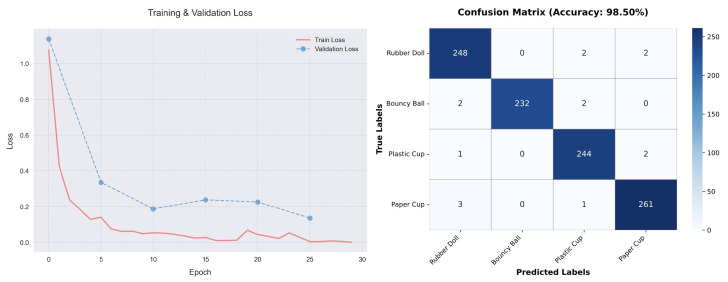
Training process and recognition test results of Tactile-Driven DCNN.

The tactile-driven object recognition module was rigorously evaluated through specialized trials. For classifying the four target objects (rubber doll, bouncy ball, plastic cup, paper cup), we implemented a streamlined DCNN architecture (detailed in Section 3.3) to balance accuracy and computational efficiency—a critical requirement for real-time deployment on the robotic mainboard. The training utilized various types of data successfully grasped in RL experiments. The protocol involved: (1) Data preprocessing: Normalizing pressure values to [0, 1] based on sensor range; (2) Optimization: Adam solver (lr = $1\times 10^{-3}$, batch size = 32) with early stopping (10-epoch patience). The model is based on PyTorch Mobile 2.5.0 for lightweight direct loading and inference. The training graph of the model and the error matrix graph of the test results are shown in the Figure 10.

Tactile-based identification achieves 98.5% classification accuracy on the test set, demonstrating the efficacy of the proposed lightweight DCNN architecture. Notably, rigid objects (plastic/paper cups) exhibit near-perfect precision due to consistent pressure signatures during initial contact. Deformable targets show marginally lower recall, attributable to transient pressure variations induced by elastic deformation.

A fascinating pattern emerges when the grasping of elastic objects is analyzed. The optimal grasp is characterized by a uniformly distributed force matrix, reflecting an equilibrium in pressure application that ensures a firm yet non-deforming grip on the object. However, the force distribution is not entirely independent of the shape of the object. In the case of an irregularly shaped doll, the pressure matrix remains evenly distributed, although its center does not align with the center of the matrix, as the fingers interact with the nonuniform surface of the object. On the other hand, for regular, spherical objects, the pressure distribution aligns closely with the center of the matrix. As the force deviates from the optimal range, significant consequences ensue; excessive force can lead to irreversible deformation of the object, which is evident in the uneven distribution of the pressure matrix. However, insufficient force does not secure a firm grip, jeopardizing the stability of the grip and risking the escape of the object from the hand.

The handling of rigid objects, exemplified by everyday objects such as cups, introduces a new layer of complexity, where the art of grasping lies in the precise modulation of force based on the properties of the object’s material. Different types of cups, such as plastic and paper cups, present varying challenges due to their differing hardness and surface textures. For smoother and more fragile plastic cups, the robotic hand must apply a moderate and evenly distributed force to ensure stability while avoiding deformation. As illustrated in the figure, when excessive force is exerted, the plastic cup, which cannot withstand pressure, undergoes deformation. In contrast, paper cups, being relatively stronger and rougher in texture, require a greater applied force to maintain a stable grip. However, beyond a critical threshold, excessive force can lead to deformation of the paper cup, with the pressure matrix showing markedly reduced values. Ensuring a uniform force distribution across all digits of the robotic hand is paramount; any deviations in force application can cause the object to warp due to excessive pressure or result in an unstable grip from insufficient force, both compromising the success of the grasp.

## 5. Conclusions

This study tackles the fundamental challenge of enabling robots to grasp geometrically and materially diverse objects in vision-denied environments, where traditional model-based approaches struggle due to their reliance on predefined object models and visual feedback. The proposed RLMP framework overcomes these limitations by embedding human-like adaptive grasping capabilities through a synergistic integration of tactile-guided reinforcement learning and novice-accessible teleoperation. Experiments have shown that RLMP achieves very small errors in human-computer interaction mapping, successfully learning to grasp different objects while achieving 98.5% classification accuracy in lightweight classifiers based solely on tactile data.

The RLMP framework addresses the fundamental challenge of enabling robots to grasp geometrically and materially diverse objects in non-visual environments, where conventional methods fail to adapt to high-precision force and contact-state requirements. By learning from human demonstrations via teleoperation, RLMP translates human grasping intuition—refined through years of biological evolution—into tactile-guided reinforcement learning policies. This allows robots to dynamically adjust finger kinematics and force profiles based on real-time pressure feedback, eliminating dependency on visual input or predefined object models. By closing the gap between human dexterity and robotic autonomy, RLMP achieves a new approach to manipulation in environments where vision is unreliable or absent.

Future work will focus on expanding RLMP’s robustness in dynamic environments: (1) addressing data drift through online incremental learning to maintain tactile recognition accuracy under sensor degradation or environmental variations (e.g., temperature/humidity changes), and (2) mitigating concept drift via adaptive reinforcement learning that dynamically updates reward functions when task requirements evolve (e.g., shifting from deformation prevention to rapid grasping). Additionally, we will explore hardware-software co-design to enhance real-time adaptability while extending the framework to collaborative tasks requiring human-robot force negotiation.

## Figures and Tables

**Table 1:** Comparison of absolute differences between joint angles of robotic arms and different mapping methods.

Joint	Absolute of Angle Difference	Mean ± Standard Deviation
Thumb Rotation angle	$|\theta_{RobotHand}-\theta_{Multimodal}|$	3.60 ± 2.16
	$|\theta_{RobotHand}-\theta_{DIP}|$	11.48 ± 5.60
	$|\theta_{RobotHand}-\theta_{Flex}|$	**3.09 ± 1.54**
Thumb bend angle	$|\theta_{RobotHand}-\theta_{Multimodal}|$	**0.43 ± 0.45**
	$|\theta_{RobotHand}-\theta_{DIP}|$	2.93 ± 2.15
	$|\theta_{RobotHand}-\theta_{Flex}|$	2.81 ± 0.85
Index bend angle	$|\theta_{RobotHand}-\theta_{Multimodal}|$	**1.34 ± 0.71**
	$|\theta_{RobotHand}-\theta_{DIP}|$	12.54 ± 7.59
	$|\theta_{RobotHand}-\theta_{Flex}|$	3.20 ± 3.54
Middle bend angle	$|\theta_{RobotHand}-\theta_{Multimodal}|$	**1.57 ± 1.14**
	$|\theta_{RobotHand}-\theta_{DIP}|$	15.47 ± 7.13
	$|\theta_{RobotHand}-\theta_{Flex}|$	6.08 ± 7.15
Thumb bend angle	$|\theta_{RobotHand}-\theta_{Multimodal}|$	**1.04 ± 0.72**
	$|\theta_{RobotHand}-\theta_{DIP}|$	8.47 ± 5.90
	$|\theta_{RobotHand}-\theta_{Flex}|$	2.75 ± 1.99
Pinky bend angle	$|\theta_{RobotHand}-\theta_{Multimodal}|$	**1.14 ± 0.99**
	$|\theta_{RobotHand}-\theta_{DIP}|$	8.36 ± 5.14
	$|\theta_{RobotHand}-\theta_{Flex}|$	3.00 ± 2.64

## Data Availability

The raw data supporting the findings of this study are available from the corresponding author upon reasonable request.
